# Rolling-element bearing vibration datasets under varying loads and speeds: A study from Vishwakarma Institute of Technology

**DOI:** 10.1016/j.dib.2025.111455

**Published:** 2025-03-13

**Authors:** Yasser N. Aldeoes, Pratibha Mahajan, Shilpa Y. Sondkar, Jitendra A. Gaikwad

**Affiliations:** a Department of Computer Engineering, Vishwakarma University, Pune, India; b Department of Artificial Intelligence, Vishwakarma University, Pune, India; c Department of Instrumentation Engineering, Vishwakarma Institute of Technology, Pune, India

**Keywords:** Vibration Data, Fault detection/Diagnosis, Machine condition monitoring, Signal processing

## Abstract

Data collection and analysis are critical for identifying and diagnosing issues in rolling-element bearings. Vishwakarma Technologies, Pune, India, has developed a unique rolling-element vibration dataset specifically gathered under controlled static load and motion conditions, adding significant value to existing public datasets. This dataset offers researchers precise vibration data that complements existing features, enabling accurate assessments of bearing conditions. Collected using accelerometers, the dataset also provides insights into bearing deterioration under sustained loads, which can help predict failures and support the development of advanced diagnostic tools. The dataset comprises 50 files that cover a wide range of operating and fault conditions, including varying motor speeds, high-loading scenarios, and both healthy and faulty bearing states. It delivers detailed, high-quality information that enhances the detection and diagnosis of rolling-element bearing problems, contributing to more reliable maintenance practices and improved system reliability.

Specifications Table

**Table:** 

Subject	Computer Engineering
*Specific subject area*	Vibration Data, Predictive maintenance, Machine condition monitoring
*Type of data*	*xlsx* (Excel file), .mat (MATLAB file)
*Data collection*	The bearing is operated from healthy to unsuccessful. This list includes internal species, external species, and healthy deficiencies. In addition, data are collected for bearing conditions at three speeds and a constant load with no load. To gather vibration data, two accelerometers were positioned in the bearing housings X and Y axes. Data was collected at three different speeds: 950, 1250, and 1950 rpm. A 1.25 kg mass and a gearbox were inserted as loads at both motor ends.
*Data source location*	Vishwakarma Institute of Technology (VIT), Bibwewadi, Pune, Maharashtra, India - 411 037
*Data accessibility*	Repository name: data.mendeley.com Data identification number: 10.17632/zgzyxdnyv9.2 Direct URL to data: https://data.mendeley.com/datasets/zgzyxdnyv9/2
*Related research article*	Aldeoes, Y.N., Mahajan, P. & Sondkar, S.Y. Advancements in Bearing Defect Diagnosis: Deep Learning-based Signal Processing and Real-time Fault Detection. J Fail. Anal. and Preven. 24, 2700–2713 (2024). https://doi.org/10.1007/s11668-024-02036-z

## 1 Value of the Data

• The dataset captures bearing performance under dynamic, variable rotational speeds, which provides a unique perspective compared to traditional datasets in the literature, focusing primarily on steady-state conditions. This characteristic makes the dataset particularly valuable for studying real-world scenarios where machinery operates under fluctuating speeds and varying loads
• Vibration data is recorded by two strategically located accelerometers in the bearing housings with one horizontally aligned and the other vertically ensuring adequate coverage of motion energy.
• Unlike conventional datasets collected under constant speeds, this dataset provides a more realistic representation of operational conditions. The inclusion of variable rotational speeds, dual accelerometer measurements (horizontal and vertical orientations), and distinct loading scenarios (1.25 kg weight and gearbox) offers a multidimensional view of bearing behaviour. These features fill critical gaps in research by enabling the development and testing of algorithms that require diverse and challenging inputs.
• The dataset provides a reliable means to evaluate the effectiveness of traditional deep learning and maintenance-related machine learning methods in detecting bearing problems under stable operating conditions. In addition, spectrograms from the dataset can be trained using to convolutional neural networks (CNNs), enabling rolling-element bearings to be identified more accurately.(Specific Applications)
• The dataset has immense potential in emerging areas like predictive maintenance and real-time fault detection. It can be used to train predictive models that anticipate bearing failures based on early warning signs, allowing timely interventions. Furthermore, its applicability in real-time monitoring systems ensures better operational efficiency and reduced downtime in industrial environments.
• The dataset's flexibility allows researchers to explore various fault detection strategies, such as hybrid methods combining traditional and machine learning approaches, and its insights can inform the design of more robust condition-monitoring systems.

## 2 Background

The dataset strengthens the value of a published article by providing reliable vibration data for rolling-element bearings under various speeds and loads. It validates fault diagnosis and predictive maintenance methods and enables the exploration of new diagnostic approaches. The dataset supports advanced signal processing techniques, such as time-frequency transformation and wavelet analysis, ensuring scalability and accuracy across diverse operating conditions. As a valuable resource for developing detection systems, it promotes transparency and collaboration among researchers and professionals. Open access fosters innovation in device state management, predictive maintenance, and industrial IoT applications. This structured dataset bridges the gap between design and implementation, driving the development of efficient maintenance strategies and encouraging the adoption of data-driven practices in industrial systems.

In this study, a systematic data acquisition and analysis process is implemented to collect, process, and store vibration data from bearings under different operating conditions. The acquired data is analyzed using MATLAB for feature extraction and pattern recognition. To ensure reproducibility and accessibility, the processed dataset is uploaded to Mendeley, enabling further research and benchmarking in the field of bearing fault diagnosis.

## 3 Data Description

The dataset [1] contains 50 files, covering different operating conditions and error conditions. These include different motor speeds, high loading conditions, and positive and faulty bearing conditions. This comprehensive design ensures that the dataset is versatile and suitable for a wide range of applications, including fault diagnosis modeling, performance simulation, and real-time status monitoring including system construction. The raw dataset consists of time-series data capturing vibrations using sensors applied to a motor operating at three different speeds (950, 1250, 1950 rpm) applied loads 1.25 kg and a gearbox (GB) at both motor ends.

**Fig. 1:**
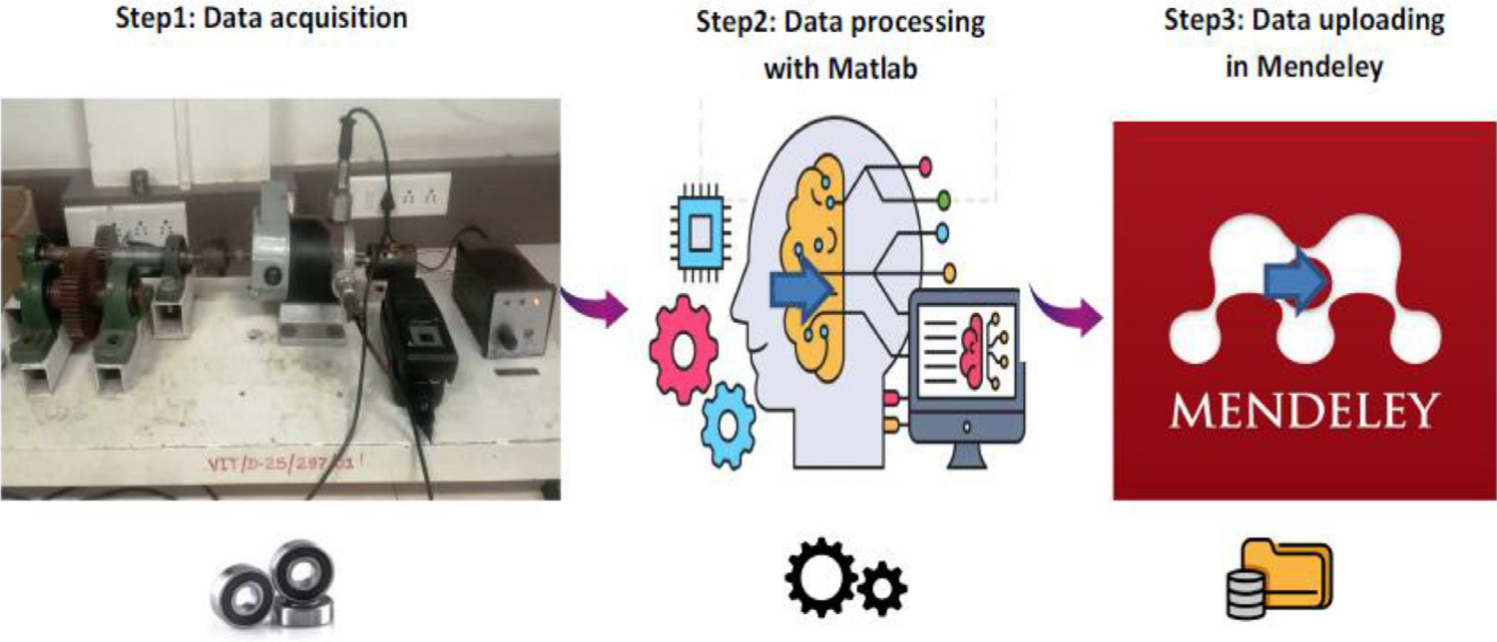
Data acquisition process.

The data acquisition process was systematically conducted through a series of well-defined steps, as illustrated in Fig. 1.

**Step 1:** The vibration data from the bearings were collected using an accumulator sensor under varying operating conditions and rotational speeds, as detailed in Table 1.

**Table 1:** Explanation of the dataset numbering system.

Fault type	File name	Loading Condition	Speed (RPM)
**Healthy Bearing**	H-NL- 950	No Load	950 RPM
	H-L- 950	Full Load (1.25kg+GB)	
	H-NL- 1250	No Load	1250 RPM
	H-L- 1250	Full Load (1.25kg+GB)	
	H-NL- 1950	No Load	1950 RPM
	H-L- 1950	Full Load (1.25kg+GB)	
**Inner Race Fault**	IRF- NL- 950	No Load	950 RPM
	IRF -L -950	Full Load (1.25kg+GB)	
	IRF- NL- 1250	No Load	1250 RPM
	IRF -L -1250	Full Load (1.25kg+GB)	
	IRF- NL- 1950	No Load	1950 RPM
	IRF -L -1950	Full Load (1.25kg+GB)	
**Outer Race Fault**	ORF –NL- 950	No Load	950 RPM
	ORF -L -950	Full Load (1.25kg+GB)	
	ORF –NL- 1250	No Load	1250 RPM
	ORF -L -1250	Full Load (1.25kg+GB)	
	ORF –NL- 1950	No Load	1950 RPM
	ORF -L -1950	Full Load (1.25kg+GB)	

**Step 2:** The acquired data undergoes processing in MATLAB, where signal analysis and feature extraction are performed to derive meaningful insights.

**Step 3:** The processed data is uploaded to Mendeley, ensuring accessibility for researchers and facilitating further analysis.

The first number in the dataset numbering identifies the condition of the bearing's health “H”, Inner race fault “IRF”, Outer race fault “ORF”. The second number specific the motor condition with load “L” and without load “NL” The third number indicts the type of the speed 950 rpm, 1250 rpm, 1950 rpm. The numbering of the dataset is given in Table 1.

To provide a clearer understanding of the collected datasets, the specific operating conditions for each dataset listed in Table 1 are detailed below. All datasets were sampled at a rate of 12,000 Hz over a 6-second period.

Dataset H-NL-950: Vibration data from a healthy bearing running at 950 rpm with no load.
Dataset H-NL-1250: Vibration data from a healthy bearing running at 1250 rpm with no load.
Dataset H-NL-1950: Vibration data from a healthy bearing operating at 1950 rpm with no load.
Dataset H-L-950: Vibration data from a healthy bearing running at 950 rpm with a load.
Dataset H-L-1250: Vibration data from a healthy bearing running at 1250 rpm with a load.
Dataset H-L-1950: Vibration data from a healthy bearing operating at 1950 rpm with a load.
Dataset IRF-NL-950: Vibration data from a faulty bearing with an internal race fault running at 950 rpm without a load.
Dataset IRF-NL-1250: Vibration data from a faulty bearing with an internal race fault running at 1250 rpm without a load.
Dataset IRF-NL-1950: Vibration data from a faulty bearing with an internal race fault running at 1950 rpm without a load.
Dataset IRF-L-950: Vibration data from a faulty bearing with an internal race fault running at 950 rpm with a load.
Dataset IRF-L-1250: Vibration data from a faulty bearing with an internal race fault running at 1250 rpm with a load.
Dataset IRF-L-1950: Vibration data from a faulty bearing with an internal race fault running at 1950 rpm with a load.
Dataset ORF-NL-950: The vibration data from a faulty bearing with an external race fault running at 950 rpm without a load.
Dataset ORF-NL-1250: The vibration data from a faulty bearing with an external race fault running at 1250 rpm without a load.
Dataset ORF-NL-1950: The vibration data from a faulty bearing with an external race fault running at 1950rpm without load.
Dataset ORF-L-950: The vibration data from a faulty bearing with an external race fault running at 950rpm with load.
The dataset ORF-L-1250: The vibration data from a faulty bearing with an external race fault running at 1250 rpm with a load.
Dataset ORF-L-1950: The vibration data from a faulty bearing with an external race fault running at 950 rpm with a load.

## 4 Experimental Design, Materials and Methods

### 4.1 Experimental design

In the context of bearing vibration analysis, experimental design involves the strategic selection of sensor placements, operating conditions, rotational speeds, and fault conditions to systematically capture meaningful data. Key considerations include sampling frequency, environmental factors, and statistical validity, ensuring the reproducibility and accuracy of fault detection studies.

**Fig. 2:**
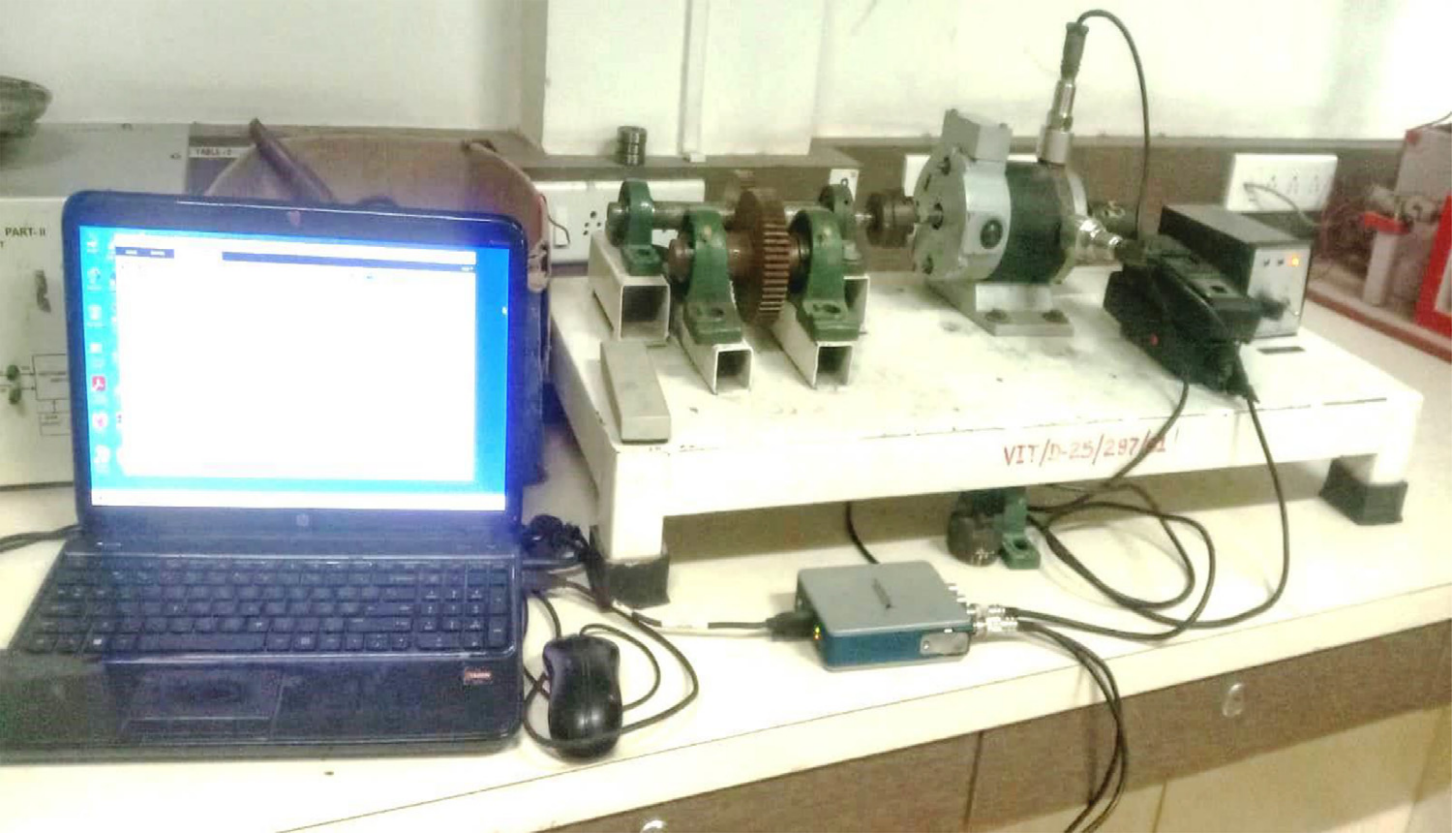
Experimental setup.

Fig. 2 illustrates the experimental setup utilized in this study. The test platform was designed for conducting bearing fault experiments, incorporating three fault modes: Healthy Bearing (HB), Outer Race Fault (ORF), and Inner Race Fault (IRF). In each experiment, a maximum of one faulty bearing is introduced, with fault bearings mounted at the motor end. To capture vibration data, two accelerometers are strategically positioned on the bearing housings in both horizontal and vertical orientations.

At both ends of the motor, two weights are applied: a gearbox and a 1.25 kg load. The testing utilized ball bearings of type 608-2RSH & SKF, as detailed in Table 2. Three datasets were gathered for each bearing fault condition (Healthy, ORF, IRF) at three motor speeds (950, 1250, and 1950 rpm), recorded for 6 seconds each at a 12 kHz sampling frequency. Faults were introduced using EDM (Outer race fault: 0.8 mm depth, 0.7 mm width; Inner race fault: same dimensions). Two piezoelectric accelerometers (100 mV/g sensitivity) were mounted horizontally and vertically on the bearing housing. Data was acquired using the NI DAQ system and saved in MAT-file format via MATLAB 2021b.

**Table 2:** Specification of bearing.

Bearing type	Contact angle	Ball count	Pitch diameter	Ball diameter	Inside diameter	Width	Outside diameter
608-2RSH&SKF	0°	8	1.537 in	0.3126 in	8mm	7mm	22mm

The experimental setup facilitates fault creation in the motor drive end bearing and ensures data collection with a minimal signal-to-noise ratio. Accelerometers were tested on both sides (x, y) of the motor casing, directly mounted on the bearing. The accelerometer positioned outside the motor casing demonstrated significantly reduced noise compared to its external placement and was unaffected by electromagnetic interference. Data was recorded exclusively with the accelerometer outside the casing, enabling researchers to analyze a clearer bearing signal with less noise. Environmental conditions were controlled at 25°C ± 2°C, with minimal ambient noise (<50 dB).

### 4.2 Materials

**Table 3:** Storage of the bearing dataset.

Sr. No.	Material	Description	Image
1	Digital Tachometer	An electronic device that measures the speed of rotation of an object, usually a shaft or wheel. It displays the speed as a digital reading, usually in revolutions per minute (RPM).	
2	Data Acquisition System (DAQ)	This device records the data from the sensor and feeds it to the laptop for analysis	
3	Bearing	Three bearing type 608-2RSH & SKF Bearing Conditions: -Outer race fault(A 0.3 mm deep, 0.5 mm wide outer race fault was created using EDM. -Healthy bearing. -Inner race fault(A 0.3 mm deep, 0.5 mm wide inner race fault was created using EDM Speeds up to 15,000 RPM.	
4	AC Drive Controller	Used to control the speed of AC motors in industrial and commercial applications	
5	Motor	The electric motor drives the rotating shaft, providing the motion necessary to generate vibration data for analysis.	
6	Vibration Sensor	**Type of Sensor:** Piezoelectric accelerometers. **Model:** Insert model number, e.g., PCB Piezotronics 352C33. **Sensitivity:** 100 mV/g. **Frequency Range:** 1 Hz to 10 kHz. **Mounting Method:** Magnetic base mounting to ensure a firm attachment and minimize noise. Calibration: Sensors were calibrated using a reference signal generator to ensure accurate readings within ±2% error margin.	
7	Gear and mass (1.25kg)	A gear and a mass of 1.25 kg are installed on both sides of the motor to function as the load during the experiment.	
8	Laptop	Used to visualize and analyze vibration data in real-time. The screen shows a waveform, likely corresponding to the vibration signals from the setup.	

#### 4.3.1 Methods

For all experiments, vibration signals were sampled at a frequency of 12,000 Hz for duration of 6 seconds under both ``Load'' and ``Unload'' conditions. A National Instruments (NI USB-6212) data acquisition device, equipped with multiple input channels, was utilized for accurate sampling and recording of analog signals. The collected data were saved in two formats: MAT (.mat) files for advanced analysis in MATLAB, and XLSX (.xlsx) files for easy access and viewing in spread sheet software. This dual-format storage ensured compatibility for both detailed computational analyses and straightforward exploratory reviews. The dataset is compatible with a wide range of deep learning and machine learning applications. While the dataset is not sufficiently large or diverse for standalone deep learning applications, it can be effectively supplemented with other publicly available datasets [2].

**Fig. 3:**
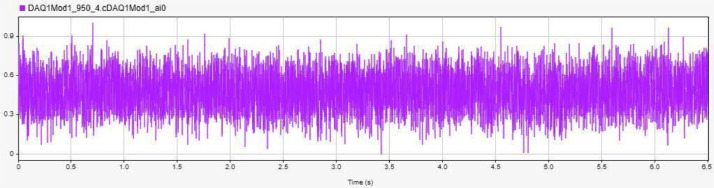
Accelerometer data for H-NL-950.

**Fig. 4:**
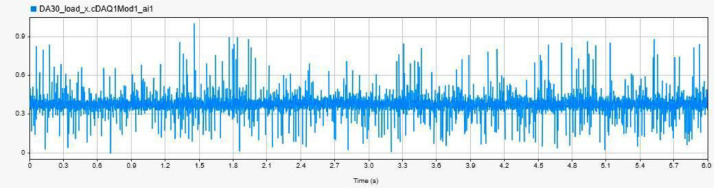
Accelerometer data for IRF-L-1250.

**Fig. 5:**
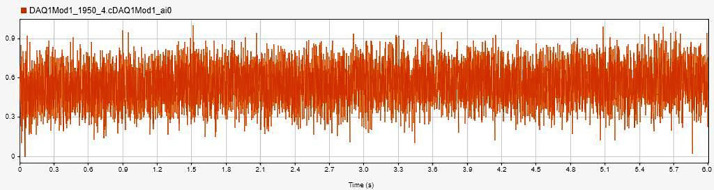
Accelerometer data for ORF-NL-1950.

Fig. 3 displays the vibration data for a healthy bearing condition under no load, labelled as `H-NL-950' In contrast, Fig. 4 showcases the vibration data for a bearing with an inner race fault under load, labelled `IRF-L-1250' while Fig. 5 presents the data for an outer race fault bearing without load, identified as `ORF-NL-1950' [3].

## Limitations

Not applicable.

## Ethics Statement

The authors confirm that they have read and adhere to the ethical requirements for publication in ‘Data in Brief’. The current work does not involve human subjects, animal experiments, or any data collected from social media platforms.

## Credit Author Statement

**Yasser N. Aldeoes:** Conceptualization, Methodology, Data Curation, Software, Writing, Review & Editing, Original Draft. **Dr.Pratibha Mahajan:** Validation, Review & Editing, Supervision. **Dr.Shilpa Y. Sondkar:** Formal Analysis, Visualization, Validation, Supervision. **Jitendra A. Gaikwad:** Formal Analysis, Installation, Visualization.

## Data Availability

Mendeley DataSkip to main content Rolling-Element Bearing Vibration Datasets under Varying Loads and Speeds: A Study from Vishwakarma Institute of Technology (Original data).
